# Lateral Unicompartmental knee arthroplasty for a secondary osteonecrosis of the lateral femoral condyle. A case report

**DOI:** 10.1186/s12891-020-03585-8

**Published:** 2020-08-31

**Authors:** Tao Yang, Huaming Xue, Tong Ma, Tao Wen, Long Xue, Mengyin Guan, Yihui Tu

**Affiliations:** Department of Orthopedic Surgery, Yangpu Hospital, Tongji University School of Medicine, Shanghai, 200090 China

**Keywords:** Lateral unicompartmental knee arthroplasty, Secondary osteonecrosis of the knee, Surgical methods, Case report

## Abstract

**Background:**

Secondary osteonecrosis of the knee is a rare event. There are few reports regarding management of this condition. The aim of the present study is to report treatment outcomes for secondary osteonecrosis of the lateral condyle treated with unicompartmental knee arthroplasty (UKA).

**Case presentation:**

A 54-year-old woman with idiopathic thrombocytopenic purpura, who received low-dosage corticosteroids, complained of knee pain for 5 years and difficulty walking in the last 5 months. Fixed-bearing lateral UKA was performed under general anesthesia combined with midthigh saphenous nerve block. The patient could walk without ambulation aid shortly after the operation and achieved satisfactory knee joint function at the 6-week follow-up. The knee society score (KSS) increased from 68 to 91. The follow-up period was up to 1 year. There was no pain, loosening, or fracture of the prosthesis at the latest follow-up.

**Conclusions:**

This case study demonstrates successful management of secondary osteonecrosis of the lateral femoral condyle is possible with a fixed bearing lateral UKA. Early diagnosis, rigorous indication, and appropriate surgical techniques were critical to maximizing prosthesis stability in lateral UKA.

## Background

Osteonecrosis of the knee was firstly described by Ahlbäck in 1968 and had been delineated into three categories: spontaneous osteonecrosis of the knee, secondary and post-arthroscopic [1, 2]. The incidence of secondary osteonecrosis of the knee is approximately 10% that of hip osteonecrosis [3]. Dissimilar to spontaneous osteonecrosis that is mainly affecting the medial femoral condyle, secondary osteonecrosis may involve both femoral condyles, as well as the epiphysis, diaphysis, and metaphysis of the involved femur and/or tibia. Often, these patients have osteonecrosis of other large joints. Recent studies reported that the femur was affected in ≤90% of cases, and > 80% of patients have bilateral disease and/or other joint involvement [2, 4]. It is more prevalent in younger patients and approximately 90% of all occurrences of secondary osteonecrosis of the knee are associated with alcohol abuse and the use of corticosteroids [3].

Unicompartmental knee arthroplasty (UKA) is considered one of the most effective treatments for knee osteonecrosis relive pain and maintain native knee kinematics [4–7]. Advantages of UKA include faster recovery, better functional outcomes, better preservation of the joint and saving of bone tissue [8, 9]. However, as it is a very rare cause of knee pain for which treatment options remain evolving, there are few reports about UKA in a patient with secondary knee osteonecrosis so far. Herein, we report a case of secondary osteonecrosis of the lateral condyle in a 54-year-old woman who was successfully treated with fixed bearing lateral UKA. The patient and her family have consented to the publication of this article.

## Case presentation

A 54-year-old woman presented at our hospital had left knee pain and gradually uncomfortable for 5 years. Symptoms rapidly worsened with limited activity in the last 5 months. The patient suffered from idiopathic thrombocytopenic purpura which needed low-dosage oral prednisone (5 mg for three times per day) 6 years ago. Recently, she was frequently suffering from pain on the lateral side of the knee during long time walk and stair performance.

Physical examination: There was focal tenderness over the lateral femoral condyle of the left knee. The physical examination elicited severely knee pain of the lateral side on extremes of range of motion, as well as during valgus stress test on the knee, but range of motion was not significantly limited. The EuroQol five-dimension (EQ-5D) quality of life score was 0.587, Knee Society score (KSS) was 64 and WOMAC score was 38.

**Fig. 1:**
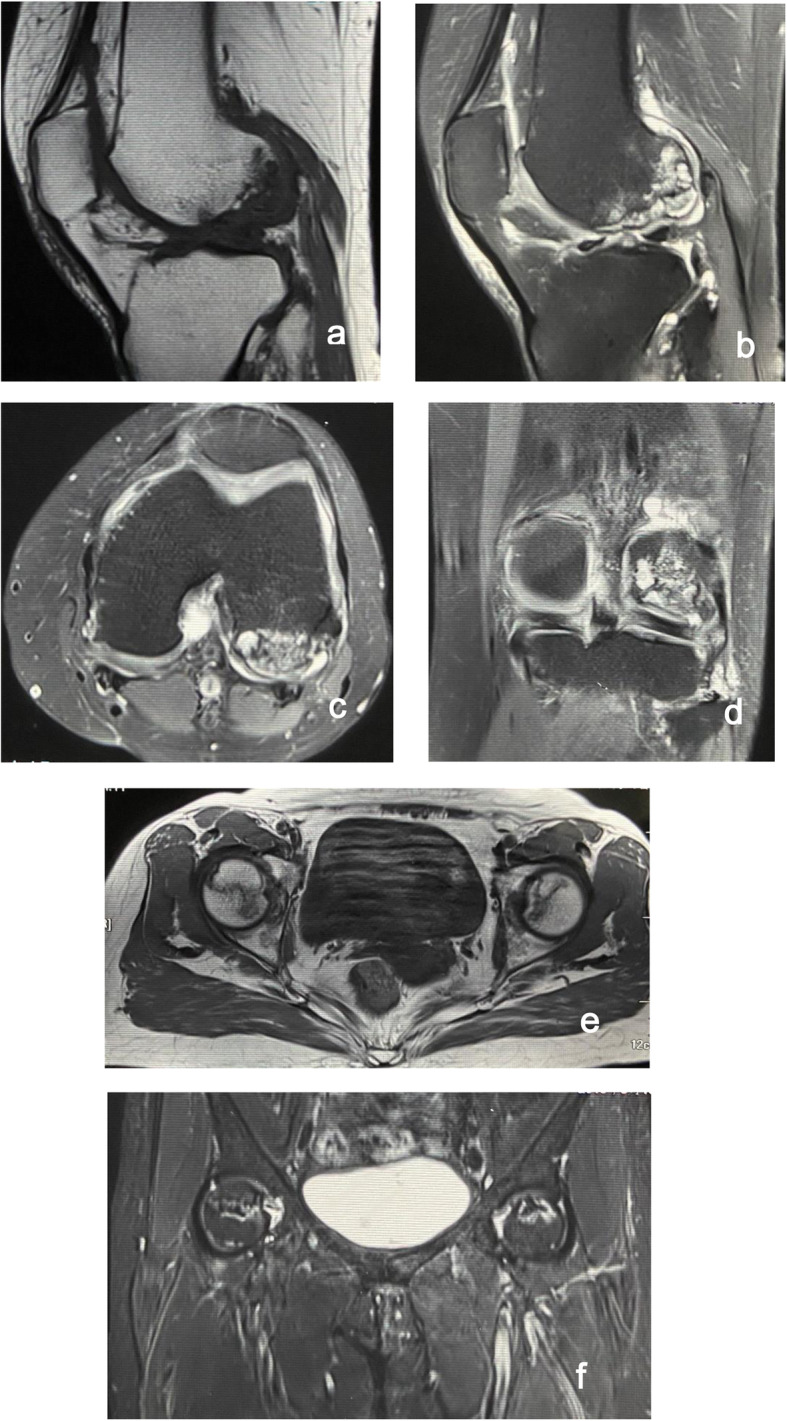
(**a**) Sagittal T_1_-weighted MRI showing a low-signal subchondral lesion of serpiginous morphology. (**b**, **c**, **d**) T_2_-weighted sequences demonstrated a serpiginous lesion of a relatively disorganized area of edema with hyperintense signal, and focal epiphyseal contour depressions. (**e**) Axial T_1_-weighted MRI showing a bilateral large subchondral lesion of isointense. (f) Coronal T-weighted MRI showing necrotic fragments in bilateral femoral head, with mixed increased and reduced signal intensity

Preoperative radiographs: Magnetic Resonance Imaging (MRI) revealed avascular necrosis of the lateral femoral condyle and bilateral femoral head necrosis (Fig. 1). According to the Ficat-Arlet classification (modified version) [2, 10], this knee osteonecrosis was classified as stage IV.

**Fig. 2:**
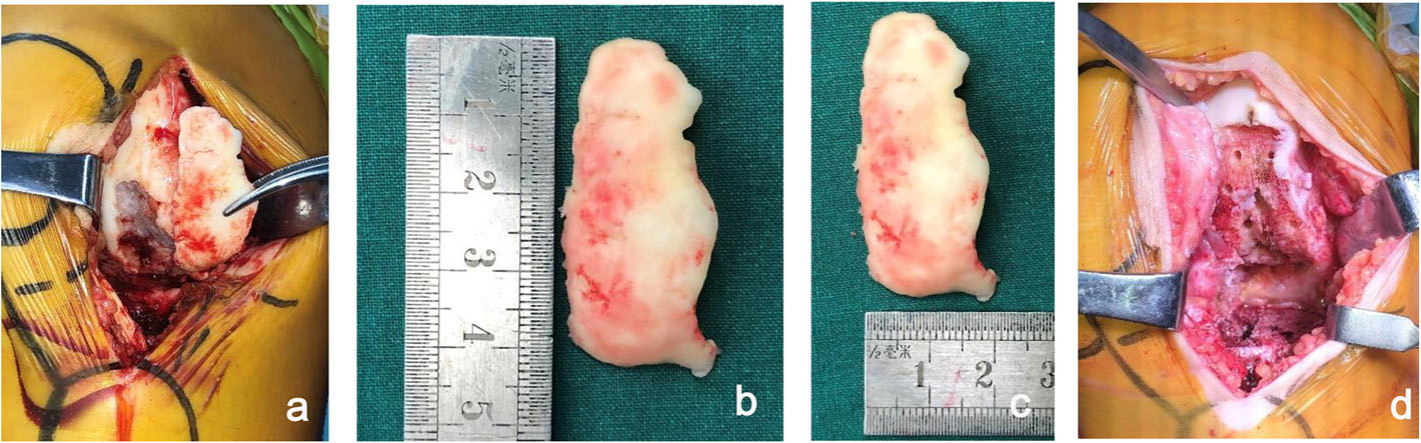
Intraoperative findings: (**a**) A large overlying cartilage over the osteonecrosis lesions. Calculation of lesion size: (**b**, **c**) The length and width of the lesion were measured in vitro (**d**) After drilling holes on the necrotic bone bed.

The diagnosis was secondary osteonecrosis of the lateral condyle and Idiopathic thrombocytopenic purpura. Due to the presence of a large lesion limited to lateral femoral condyle, no evidence of joint space narrowing in the medial tibia-femoral compartment, and intact cruciate and collateral ligaments, the therapeutic treatment was fixed bearing lateral unicompartmental knee arthroplasty (LINK German). General anesthesia combined with midthigh saphenous nerve block was used for the operation. The knee was exposed via a lateral parapatellar approach to achieving a good view. Osteonecrosis of the distal aspect of the femur produces a large segment of dead bone (approximately 8.17 cm^2^) on the weight-bearing portion of the lateral femoral condyle (Fig. 2). A large amount of necrotic bone, which mainly located on the posterior portion of femoral condyle, was completely removed down to the bleeding bed of bone by a spatula. Then we drilled several holes on the necrotic bone bed (Fig. 2) and filled the large bone defect with cement, which facilitated a solid initial fixation for cemented prosthesis. The rest of procedures was performed according to the lateral UKA operation manual. After the surgery, we enjoined her to avoid excessive knee flexion and intense activity in the early stage after surgery.

**Fig. 3:**
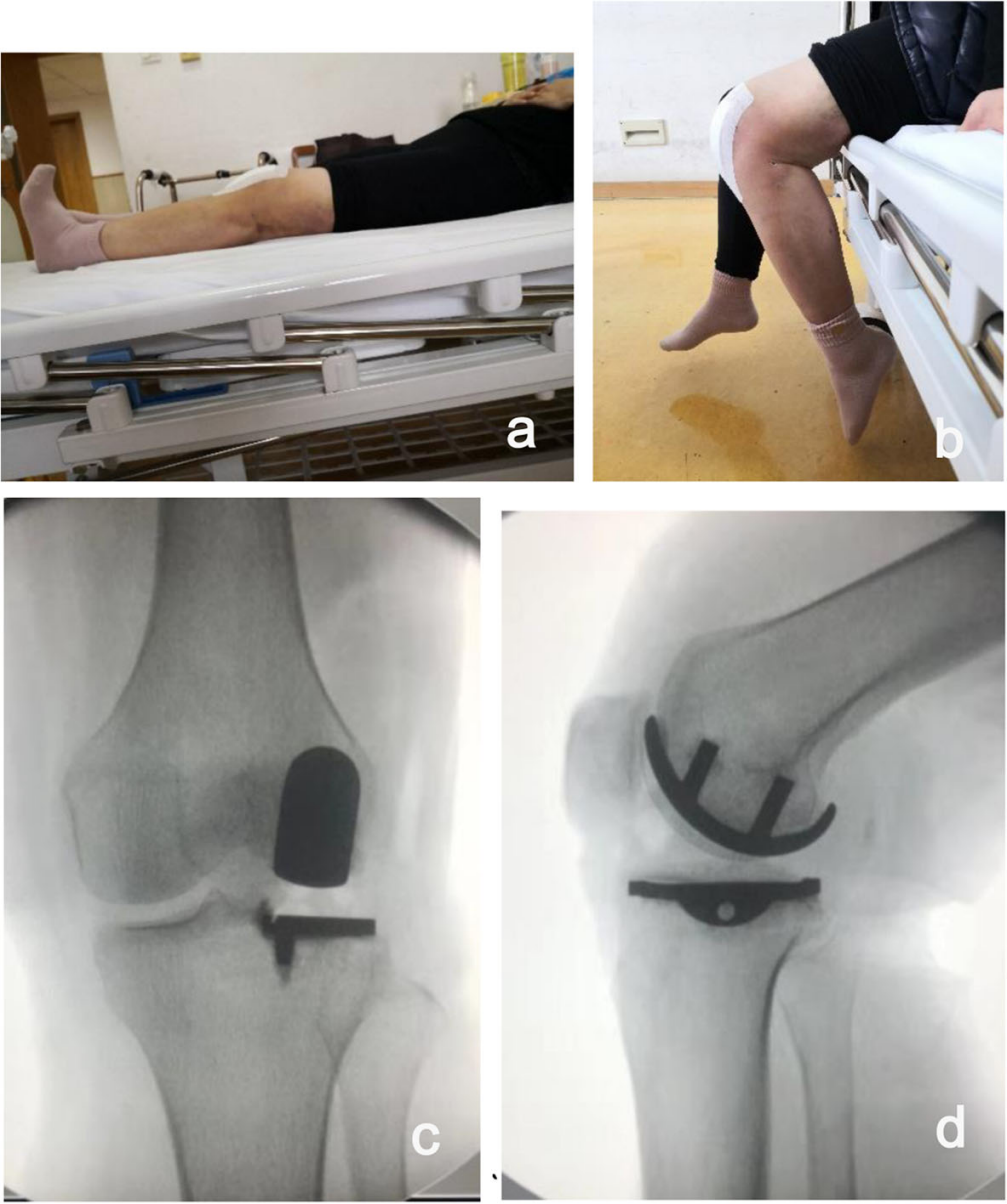
(**a**, **b**): Postoperative clinical functions: range of motion was 0° to 90°. (**c**, **d**) Postoperative anteroposterior and lateral radiographs showing an optimal size and precise position of the prosthesis

Follow-up: The patient was evaluated clinically and radiographically at 6 weeks, 3 months, 6 months and 1 year postoperatively and on an annual basis thereafter unless a problem arose. She could walk without ambulation aid shortly after the operation on the surgery day by virtue of rapid anesthetic resuscitation from general anesthesia combined with midthigh saphenous nerve block. Postoperative radiographic imaging showed optimal size and precise position of the prosthesis. On the 2nd day after surgery, she felt significant pain relief and VAS pain score improved from 7 to 2. Her left knee range of motion (ROM) was at 0° to 90° (Fig. 3). The patient could unlimitedly walk for hours, go up and down stairs freely and achieved satisfactory knee joint function with ROM of 0° to 120° at 6 weeks after the operation. Improving joint function provided a physical, mental and emotional boost to the patient. She could return to work and sport at two months postoperatively. The EQ-5D score was 1, the KSS and WOMAC score were 91, 20 respectively at the latest follow-up. The total follow-up period was 1 year and there was no pain, loosening, fracture, or wear of the prosthesis.

## Discussion and conclusions

Knee osteonecrosis is an uncommon disease that is initially managed non-operatively or with joint-sparing surgical procedures [3, 11, 12]. Secondary osteonecrosis of the knee has a multifactorial etiology and is characterized by loss of bone blood circulation. The main mechanisms may include elevated intraosseous pressure resulting from adipocyte hyperproliferation, bone death and vascular occlusion in subchondral bone caused by fat emboli [13]. Corticosteroid use is one of the most common risk factors associated with secondary knee osteonecrosis. Interestingly, we observed that the onset of knee pain occurred approximately 1 year after low-dosage prednisone administration and the symptom rapidly progressed to a worsened situation in a short period. The differential diagnosis may be challenging. MRI has become the gold-standard imaging modality to diagnose osteonecrosis. Bone infarcts and a serpiginous lesion surrounded by the characteristic double-line sign of both low and high signal could be observed on MRI [14]. Moreover, determining the onset and early diagnosis is crucial to the treatment of knee osteonecrosis as the presence of a subchondral area of low T1WI signal > 4 mm thick strongly predicts irreversibility [15].

Secondary osteonecrosis of the lateral condyle is rare compared to lateral unicompartmental osteoarthritis of the knee, and it is much more challenging to treat. Joint arthroplasty is the most appropriate surgical option when the severe subchondral collapse occurred. Although total knee arthroplasty (TKA) is suggested in patients with secondary osteonecrosis [3, 16], several studies have demonstrated excellent results of UKA treating for advanced-stage osteonecrosis of the knee [17–20]. Parratte S et al. [21] found that there was a 12-year Kaplan-Meier survivorship of 96.7% in 30 patients with osteonecrosis at a minimum follow-up of 3 years, including 10 UKAs in patients with secondary osteonecrosis. Similarly, Marmor [22] reported 2 cases of secondary osteonecrosis treated with a UKA out of a group of 34 knees and the results were successful. Therefore, secondary osteonecrosis of the knee should not be considered as contraindication for UKA.

Lateral UKA is conventionally regarded as appropriate for patients with lateral unicompartmental osteoarthritis of the knee [23]. In fact, Lateral UKA constitutes only 5–10% of all unicompartmental arthroplasty performed and represents less than 1% of all knee arthroplasty procedures [24, 25]. Notably, The use of UKA should be restricted to patients with secondary osteonecrosis of the knee, as this entity typically involves the metaphyseal region and both compartments [3, 4]. Despite the classic multifocal expression of secondary osteonecrosis, the present case met all the inclusion criteria. Obviously, lateral UKA should be considered as a reasonable surgical option for the 54-year-old woman. Similarly, Argenson JN [26] demonstrated that UKA was chosen as a less invasive procedure than TKA for patients presenting with steroid-induced osteonecrosis within a general disease context. However, contraindications in UKA due to lateral condyle osteonecrosis, to our knowledge, include osteonecrosis involved the medial compartment and excessive bone defects resulted from oversized lesion limited to lateral compartment. During the surgery, we observed the large lesion was similar to osteochondritis dissecans and the cartilage layer of the lateral femoral condyle completely detached from the bone. Under the cartilage layer, the necrotic area was larger and more diffuse than the segmental necrosis observed in idiopathic osteonecrosis of the medial femoral condyle. Besides, full-thickness cartilage was preserved at medial isolated compartment, combined with intact cruciate and collateral ligaments. Due to the rarity of secondary osteonecrosis of the lateral condyle, there was nearly no experience for reference. Therefore, how to achieve a stable joint prosthesis after thoroughly removing the necrotic bone remained the most challenges in such situations.

The present case report provides meaningful information to surgeons. Firstly, using of low-dosage corticosteroids (prednisone) could result in osteonecrosis of both knee and bilateral femoral head in a short period. The onset of the disease is more gradual at the beginning and it became rapidly progressive subsequently. Secondly, evolving into massive osteonecrosis focus on lateral femoral condyle is rare but could occur in secondary knee osteonecrosis. Our case exhibited appropriate surgical technique of cementation dealing with extensive bone defect could contribute to satisfactory radiographic outcomes. Although the patient felt significant pain relief and achieved satisfactory knee joint function, it is essential to avoid excessive knee flexion, squat and intense activities in the early period after surgery. Similarly, B. P. Chalmers et al. [6] analyzed outcomes of 46 UKAs for osteonecrosis involving an isolated compartment of the knee and found Survivorship free of any revision in the cohort was 89% (95% CI 77 to 99) and 76% (95% CI 53 to 99) at five and ten years, respectively. No implants were revised for loosening, fracture, or wear. Therefore, compared to TKA, lateral UKA is an alternative option for the management of large osteonecrosis of lateral femoral condyle in the young and active patient. Lastly, it is the first report the lateral UKA was performed under general anesthesia combined with midthigh saphenous nerve block. In fact, adductor canal block was often performed in TKA to relieve postoperative pain and provide better effectiveness of early rehabilitation [27]. Enrique A et al. [28] had reported the median hospital stays of a total of 55 patients, who treated with TKA receiving a spinal anesthetic combined with adductor canal block and periarticular injection was 25.8 h. Hence, it is possible to modify anaesthetize mode on surgical procedures for adult UKA, especially outpatients in future.

In conclusion, clinicals should be aware of secondary osteonecrosis of the lateral condyle in the patient receiving corticosteroids. In addition, our case demonstrated successful lateral UKA performing on secondary osteonecrosis of the lateral condyle, showing both excellent clinical and radiographic outcomes at 1-year follow-up. Early diagnosis, rigorous indication, and appropriate surgical techniques were critical to maximizing prosthesis stability in lateral UKA. Although this procedure is technically challenging, we wish our case can provide references for clinical treatment on the similar situations. However, given the rarity of the case and the follow-up time is not long enough, there still need further studies with great numbers and long-term follow-ups of survivorship in the patient.

## Data Availability

The datasets used and/or analysed during the current study are available from the corresponding author on reasonable request.

## Abbreviations

UKA: unicompartmental knee arthroplasty
KSS: knee society score;
MRI: Magnetic resonance imaging
ROM: range of motion
TKA: total knee arthroplasty
